# Characteristics of new and re-initiating antiretroviral therapy clients in Malawi: implications for client counseling in resource-constrained settings

**DOI:** 10.1080/16549716.2026.2740360

**Published:** 2026-10-06

**Authors:** Marguerite Thorp, Kelvin Balakasi, Isabella Robson, Colbert Lemani, Sam Phiri, Risa Hoffman, Morna Cornell, Kathryn Dovel

**Affiliations:** a Division of Infectious Diseases, University of California, Los Angeles, CA, USA; b Implementation Science Department, Partners in Hope, Lilongwe, Malawi; c Centre for Integrated Data and Epidemiological Research (CIDER), School of Public Health, University of Cape Town, Cape Town, South Africa

**Keywords:** HIV, adherence counseling, interruption in treatment, patient retention, differentiated service delivery

## Abstract

New antiretroviral treatment (ART) initiates and re-initiators (returning after interruption in treatment [IIT], defined as ≥28 days late for refill) are at higher risk of future IIT compared to stable clients. Counseling is a core strategy to increase retention among new initiates, but little is known about whether re-initiators may have different counseling needs. We compared sociodemographic and psychosocial characteristics of new initiates and re-initiators. We enrolled ART clients ≥15 years living in nine facility catchment areas across three regions of Malawi. We conducted a survey with domains on sociodemographics, ART history, mobility, depression (positive screen: ≥8 on WHO Self-Reporting Questionnaire), and alcohol use (hazardous use: ≥4 for men, ≥3 for women on AUDIT-C). We used Wilcoxon rank-sum and chi-squared tests to assess differences between new initiates and re-initiators. Between September 2023 and April 2025 we screened 1,332 and enrolled 840 eligible ART clients (239 new initiates [28%]). Median age was 38 years; 61% were female. New initiates were more likely than re-initiators to be younger (33 vs. 39 years, *p* < 0.01), pregnant (21% vs. 4%, *p* < 0.01), unaware of spouse’s HIV status (63% vs. 4%, *p* < 0.01), screened positive for depression (48% vs. 26%, *p* < 0.01), and reporting high-risk alcohol usage (34% vs. 19%, *p* = 0.03). New initiates were less likely to be mobile (21% traveled ≥30 nights in last 12 months vs. 60%, *p* < 0.01). Counseling should address different needs for new vs. re-initiating clients, targeting depression, alcohol use and disclosure among all clients, disclosure and DSD models for new initiates, and mobility for re-initiators.

## Paper Context

**Main findings**: Clients who were newly initiating antiretroviral treatment were less likely to know their spouse’s HIV status and to be mobile than those reinitiating treatment, but both groups reported high rates of depression and high-risk alcohol use.

**Added knowledge**: This study compared clients who were initiating antiretroviral treatment for the first time with those who were reinitiating after an interruption in treatment, two populations that are sometimes grouped together despite very different needs.

**Global health impact for policy and action**: Counseling for psychosocial needs and alcohol reduction may be needed by both new and reinitiating clients, while re-initiators may need more counseling about mobility and less about disclosure and differentiated service delivery models.

## Background

As national antiretroviral therapy (ART) programs surpass UNAIDS’ 90–90–90 goals, achieving 95–95–95 targets and epidemic control will require increased retention in care for people living with HIV (PLHIV) [1]. In the context of limited resources, programs must strategically target and tailor retention services to maximize impact.

Client counseling has been a core strategy in increasing retention [2], but counseling curricula focus almost exclusively on biomedical aspects of ART and the importance of daily pill-taking, with little regard for clients’ unique barriers or previous history with ART services [3]. In many settings, including Malawi, national guidelines recommend reiterating the standard counseling offered to new clients when an ART client returns after an interruption in treatment (IIT) (defined as ≥28 days late for an ART appointment) [4]. Enhanced adherence counseling (EAC) is prescribed for returning clients – at least one session is mandated by Malawi’s national guidelines – but the content of EAC is not clearly differentiated from the curriculum designed for new clients. Though some supporting organizations encourage person-centered care that focuses on individual client needs, counselors are given little or no formal guidance on which topics may be more suitable for different client groups.

As resources for person-centered care dwindle due to declining bilateral aid, counselors must offer support efficiently to cope with increasing workloads. Heuristics about the different needs of easily identifiable client groups, such as new versus re-initiating clients, may help counselors provide effective tailored counseling quickly. We examined sociodemographic, psychological, and health service characteristics of new and returning ART clients in Malawi to identify patient characteristics that may inform tailored HIV counseling strategies.

## Methods

This cross-sectional analysis used survey data from new and reinitiating (returning after IIT) ART clients at nine health facilities in Malawi. Clients were identified in health facilities on the same day they started ART or re-started ART after missing an appointment by ≥28 days. Other eligibility criteria included age ≥ 15 years and living in facility catchment area. Research assistants administered a survey upon enrollment that included sociodemographic characteristics, ART history and knowledge, and questions on risk factors for treatment interruption including alcohol use, depression, and mobility. We used chi-squared tests and Wilcoxon rank‑sum tests with a significance threshold of *p* < 0.05 to identify differences between new and returning ART clients. IRB approval was obtained from Malawi’s National Health Sciences Research Council (#23/04/405) and the University of California Los Angeles (#IRB-23-5105) and all study activities complied with the Declaration of Helsinki. Written informed consent was obtained by trained research assistants on the day of enrollment for participants 18 years of age and older, and informed assent with informed consent of a guardian was obtained for participants 15–17 years of age.

## Results

We conducted surveys with 840 clients between September 2023 and April 2024: 239 new initiates and 601 ART clients returning to care (Table 1). New initiates were younger (median age 33 vs. 39 years) and more likely to be pregnant (21% vs. 4%), consistent with appropriately high HIV testing rates in Malawi’s antenatal clinics. Clients did not differ with regards to the educational attainment or economic indicators we tested.

**Table 1.** Characteristics of clients initiating and re-initiating art in Malawi, Sept. 2023–Apr. 2024 (*N* = 840).

	Total (*N* = 840)	New initiators (*n* = 239)	Re-initiators (*n* = 601)	p-value
SOCIODEMOGRAPHICS				
Age, median (IQR)	39 (30–46)	33 (26–42)	39 (31–47)	<0.01 **
Gender, % women	517 (62%)	154 (64%)	363 (60%)	0.28
Currently pregnant, %	49 (9%)	33 (21%)	16 (4%)	<0.01 **
Married, %	477 (57%)	146 (61%)	331 (55%)	0.11
Married & partner unaware of participant’s HIV status, %	106 (23%)	92 (63%)	14 (4%)	<0.01 **
Top wealth quintile, %	155 (18%)	49 (21%)	106 (18%)	0.33
DEPRESSION				
Depression screening positive (SRQ ≥ 8), %	317 (38%)	114 (48%)	203 (34%)	<0.01 **
ALCOHOL USE				
High-risk alcohol use per AUDIT-C, %	179 (21%)	63 (26%)	116 (19%)	0.02 *
ART KNOWLEDGE				
Has heard of emergency refills, %	631 (75%)	154 (64%)	477 (79%)	<0.01 **
Has heard of 6-month MMD, %	686 (82%)	149 (62%)	537 (89%)	<0.01 **
Ever received 6-month MMD, %			337 (56%)	
MOBILITY IN LAST 12 MONTHS				
Total nights away, median (IQR)	30 (2–62)	3 (0–21)	37 (10–81)	<0.01 **
Took at least one trip ≥ 30 nights, %	408 (49%)	49 (21%)	359 (60%)	<0.01 **
Took a trip that lasted longer than expected, %	461 (55%)	53 (22%)	408 (68%)	<0.01 **
REASONS FOR MISSED APPOINTMENT				
Traveling for non-work purpose, %			234 (39%)	
Traveling for work, %			103 (17%)	
Forgot, did not want to go, or no reason, %			92 (15%)	
Other obligations (social or work) without travel, %			65 (11%)	
Illness, %			37 (6%)	
Financial barrier (e.g. lack of transport funds), %			23 (4%)	
Fear of disclosure or negative provider experience, %			12 (2%)	
Other, %			35 (6%)	

*p < 0.05; **p < 0.01.

Our findings suggest that re-initiators may require different counseling than new initiates. Disclosure is a common topic in counseling curricula, for example, which is appropriate for new initiates, as 63% were married with a partner who is unaware of their HIV status; however, only 4% of re-initiators’ primary partners did not know their HIV status. We also found high rates of awareness of differentiated service delivery (DSD) models (emergency refills at health facilities while away from home and 6-month dispensing) in both groups, and higher among re-initiators (79% vs. 64% and 89% vs. 62% respectively). Of note, the high rates of DSD awareness even among new initiates suggest that initial counseling sessions, which were conducted shortly before the survey was administered, are effective in communicating common DSD models; awareness is even higher among re-initiators, suggesting that re-initiation counseling need not dwell on DSD models.

Psychosocial needs were high in both groups. New initiates were more likely to screen positive for depression and high-risk alcohol use (48% and 26% respectively), although both risk factors were also prevalent among re-initiators (34% and 19% respectively).

There were stark differences between groups in their mobility history. Compared to new initiates, re-initiators traveled more in the last 12 months (median 37 nights [IQR 10–81] vs. 3 [IQR 0–21]), had longer trip durations (60% took a trip ≥30 nights vs. 21% of new initiates), and reported more unpredictable travel (68% took a trip that lasted longer than expected vs. 22% of new initiates). These results are consistent with what we know about IIT in Malawi: mobility is the most common reason for missing appointments, both in these participants and in other studies [5], so clients returning after a missed appointment are likely to report recent high-risk mobility.

## Conclusion

These findings yielded several insights. First, psychosocial support and counseling on alcohol reduction is important for all clients; though new initiates were more likely to screen positive for depression and high-risk alcohol use, rates were high in both groups. Second, counseling curricula that require time and resources discussing spousal disclosure or DSD models may be essential for new initiates but are less likely to benefit re-initiating clients.

Finally, regarding mobility, clients returning after mobility-associated IIT should be able to discuss with counselors why they missed an appointment and strategies for avoiding repeat IITs in the future. This is an important element of person-centered care. In light of our findings, client counselors should recognize mobility as a leading cause of IIT and focus on mobility-specific counseling topics, if appropriate. However, new clients may also benefit from anticipatory, mobility-specific counseling to avoid future IITs. This seems especially true if they report recent high-risk travel, as was the case for 1 in 5 new initiates in our sample.

As national ART programs seek to increase retention despite dwindling resources, providing the right counseling will be essential. Understanding which client groups should be offered specific counseling topics may save time and resources at the facility level. In Malawi, we found that psychosocial and alcohol reduction counseling may be needed by both new and reinitiating clients, while counseling on disclosure and DSD models may be de-emphasized for re-initiators – perhaps freeing time for mobility-specific counseling for those most at risk of mobility-associated IIT.

## Data Availability

The data that support the findings of this study are available on request from the corresponding author, MT. The data are not publicly available due to the sensitive nature of HIV-related data and potentially identifying information.
